# Ultra-Low-Power, High-Accuracy 434 MHz Indoor Positioning System for Smart Homes Leveraging Machine Learning Models

**DOI:** 10.3390/e23111401

**Published:** 2021-10-25

**Authors:** Haq Nawaz, Ahsen Tahir, Nauman Ahmed, Ubaid U. Fayyaz, Tayyeb Mahmood, Abdul Jaleel, Mandar Gogate, Kia Dashtipour, Usman Masud, Qammer Abbasi

**Affiliations:** 1 Department of Electrical Engineering, University of Engineering and Technology, Lahore 54890, Pakistan; haq.nawaz@uet.edu.pk (H.N.); nahmed@uet.edu.pk (N.A.); ubaid@uet.edu.pk (U.U.F.); tayyeb@uet.edu.pk (T.M.); 2 James Watt School of Engineering, University of Glasgow, Glasgow G12 8QQ, UK; Qammer.abbasi@glasgow.ac.uk; 3 Department of Computer Science, Rachna College of Engineering and Technology (RCET), University of Engineering and Technology, Lahore 54890, Pakistan; abduljaleel@uet.edu.pk; 4 School of Computing, Edinburgh Napier University, Edinburgh EH10 5DT, UK; m.gogate@napier.ac.uk; 5 Department of Electronics Engineering, University of Engineering and Technology, Taxila 47080, Pakistan; usman.masud@uettaxila.edu.pk

**Keywords:** indoor positioning system (IPS), time difference of arrival (TDOA), ultra-low power, telemetry link

## Abstract

Global navigation satellite systems have been used for reliable location-based services in outdoor environments. However, satellite-based systems are not suitable for indoor positioning due to low signal power inside buildings and low accuracy of 5 m. Future smart homes demand low-cost, high-accuracy and low-power indoor positioning systems that can provide accuracy of less than 5 m and enable battery operation for mobility and long-term use. We propose and implement an intelligent, highly accurate and low-power indoor positioning system for smart homes leveraging Gaussian Process Regression (GPR) model using information-theoretic gain based on reduction in differential entropy. The system is based on Time Difference of Arrival (TDOA) and uses ultra-low-power radio transceivers working at 434 MHz. The system has been deployed and tested using indoor measurements for two-dimensional (2D) positioning. In addition, the proposed system provides dual functionality with the same wireless links used for receiving telemetry data, with configurable data rates of up to 600 Kbauds. The implemented system integrates the time difference pulses obtained from the differential circuitry to determine the radio frequency (RF) transmitter node positions. The implemented system provides a high positioning accuracy of 0.68 m and 1.08 m for outdoor and indoor localization, respectively, when using GPR machine learning models, and provides telemetry data reception of 250 Kbauds. The system enables low-power battery operation with consumption of <200 mW power with ultra-low-power CC1101 radio transceivers and additional circuits with a differential amplifier. The proposed system provides low-cost, low-power and high-accuracy indoor localization and is an essential element of public well-being in future smart homes.

## 1. Introduction

The process of determining the location of any object using wireless technologies is called radiolocation or wireless localization [1]. There is a high demand for wireless localization and positioning systems that can provide precise and accurate positioning for both indoor and outdoor environments in future smart homes. Recently, indoor positioning systems have emerged as a very popular indoor localization technology. The indoor positioning system should provide a precise position inside a closed environment where a complex wireless propagation environment exists due to multipath effects [2,3].

Wireless indoor positioning systems have been successfully used in many indoor applications, such as target tracking, inventory management, etc. Such wireless indoor positioning systems can be used for localization; tracking or monitoring applications where Global Positioning System (GPS) based solutions are not feasible [4]. Indoor positioning systems can provide automatic location information for different objects in many indoor scenarios. Indoor positioning systems are at the core of location-based services [5], because such applications use the subscriber’s location to provide navigation, localization and healthcare services, etc. The accuracy of deployed localization systems directly affects the performance and reliability of location-based services [6]. Indoor positioning systems can provide different types of location information, for example, in the form of coordinates, absolute location, relative location, etc. Various wireless technologies are used for wireless indoor positioning systems, ultimately providing the distance moved by a wireless transmitter with respect to reference receivers. Wireless localization systems can be based on a single technology, such as WiFi, Bluetooth, radio frequency identification (RFID), etc., or they can use hybrid topologies like the combination of WiFi and Bluetooth. A detailed comparison of different localization and positioning technologies is presented in [7]. This comparison provides information regarding the accuracy, installation cost, advantages, disadvantages, and system complexity of various indoor positioning systems (IPSs) which are based on different technologies.

Wireless indoor positioning systems that locate an active wireless transmitter with the help of information extracted from the received signal of a target transmitter use three main techniques for localization: triangulation (lateration and angulations) [8], received signal strength scene analysis [9,10], and proximity detection [11]. Triangulation uses the geometric properties of triangles to detect the location of the wireless transmitter. Lateration and angulations are two versions of triangulation used to locate the wireless transmitter. Lateration is a range measurement technique which extracts the position of an object by determining its distances from multiple reference receivers. Normally, Time of Arrival (TOA) or Time Difference of Arrival (TDOA) measurements are used to calculate the required distances of the wireless transmitter from the reference receivers [8]. Some implemented systems use round trip time of flight (RTOF) [12] for object localization. Angulations techniques use relative angle information from the reference receivers to estimate object’s position [13,14,15,16]. Some implemented systems [15] use a combination of TOA and Angle of Arrival (AOA), while others use received signal strength indication in combination with AOA techniques for indoor localization [16]. In [17], a TOA estimation technique was presented that was based on an iterative cleaning mechanism to extract the low-power first path signal where the conventional matched filtering-based TOA estimator was unable to achieve good accuracy. However, acoustic ranging was less effective, especially in noisy environments.

In addition, the ultra wideband (UWB) technique is a promising technology for indoor positioning systems, as it mitigates multipath issues due to possessing a wider bandwidth [18,19]. However, UWB systems are expensive, which results in increased cost of localization systems [19]. Moreover, various wireless systems including WiFi, Bluetooth and Zig-Bee have been exploited for positioning with good accuracy, along with data transfer capabilities [20]. However, such systems provide good ranging accuracy in the case of line-of-sight scenarios, provided that wider bandwidth is used. Moreover, these localization techniques are dependent on the coverage of such wireless networks in the specified localization area. Some indoor localization systems provide simultaneous localization and mapping by estimating information on the basis of the measured ambient magnetic fields existing in all indoor environments. The localization accuracy of such systems is dependent on the measured magnetic fields in multiple directions, rather than the measurement of the strength of the magnetic field only [21].

Apart from indoor localization techniques that are based on radio propagation characteristics (time, phase and signal strength, etc.), channel characteristics like channel state information (CSI) can also be exploited for localization [22,23]. However, the traditional CSI-based localization techniques are based on statistical parameters extracted from the individual subcarrier, and inter-dependence of adjacent subcarriers is not considered, which may result in loss of critical localization-related information. The system complexity is increased when the information related to the relation between adjacent subcarriers is quantified to estimate the position of the target [23].

All of the localization techniques stated above have certain advantages and limitations for specific localization problems, as detailed in Reference [24]. However, TDOA-based localization techniques have emerged as being very useful and effective due to the availability of low-cost, low-power and compact commercial radio receivers to realize less expensive and more power-efficient localization systems integrating data transfer capabilities [25]. The proposed indoor positioning system here in our work is based on the TDOA technique; as discussed in the next section.

The performance of indoor positioning systems can be evaluated using several metrics [26]. The accuracy or the location error performance metric is determined by calculating the error distance between the estimated position by system and the actual or true position of target. There is often a trade-off between accuracy and other parameters of the positioning system. The accuracy of the system is determined only by calculating the error distance between the true location and the estimated location, but location precision considers the variation in the measured or estimated location results over time. The positioning system is considered to be more precise if it provides very close results for the same location in each trial under the same test and measurement conditions. Localization systems with low power consumption are preferable, because such systems can be powered by batteries, offering freedom of mobility.

Several indoor wireless positioning systems based on TDOA techniques have been implemented, as reported in References [27,28,29,30,31,32,33,34,35]. Normally, a TDOA-based localization technique requires synchronization between measurement nodes in order to improve the accuracy of localization [29,31,35]. However, some implemented systems have accomplished the localization task without the need for clock synchronization [28,30]. Some such systems exploit correlations using wideband signals or wire connections between measuring units to synchronize them, but these approaches result in additional complexity. In Reference [27], different strategies for the placement of reference receivers for TDOA-based localization systems were presented, and a new spherical code approach was employed. This described the influence of the reference receivers’ placement on the accuracy of TDOA-based positioning systems. An efficient localization algorithm was proposed in [28] using TDOA without synchronization between the measuring units. The TDOA equations were performed by continuously changing the position of the target and the measuring units. The performance of the proposed algorithm was enhanced using a total least squares (TLS) technique.

The time synchronization was improved in Reference [29] in order to enhance the positioning accuracy of TDOA-based location estimation techniques. A compensation algorithm was used to reduce the time synchronization to within 5 ns, showing an increase in the location estimation system performance by 24.2% on average. A scheme called Whistle was presented in Reference [30] for TDOA-based localization without time synchronization of the measuring nodes. Several asynchronous receivers were used to record a target signal and a successive artificially generated signal. The high time resolution was achieved using sensing and sample counting techniques for the recorded and artificially generated signals. In Reference [31], a TDOA positioning method using three receivers and knowledge of some of indoor features (reflective surfaces, etc.) was presented and tested, and was able to estimate the transmitter’s location with better accuracy than the conventional TDOA schemes. A novel positioning algorithm for use in non-line-of-sight (NLOS) scenarios was proposed in Reference [32]. The proposed scheme used the geometry of the radio propagation paths to estimate the location of the target. This was based on TDOA, angle of departure (AOD) and angle of arrival (AOA) information. The performance of the proposed iterative localization algorithm based on linearization was analyzed using indoor localization measurements. The TDOA-based localization system presented in Reference [33] employed multiple surface beacons to transmit positioning messages to the receiver node, which was located under the surface of the water. The presented system computed the location of the mobile node without time synchronization between the surface beacons and the underwater mobile node.

In Reference [28], the performance and comparative analysis of several existing TDOA-based localization techniques were discussed with respect to the presence of positioning errors in the sensors. Simulation results were presented to compare the positioning accuracy of such TDOA-based localization methods in the case of high levels of positioning errors in the employed sensors. In Reference [34], a new technique based on four beacons was used to solve the three-dimensional TDOA problems in a sensor network. It was shown that the localization algorithms could be used 96.7% of the time for vehicles moving at velocity of less than 25 m/s. The Least Squares (LS) method using the Chan algorithm and the Taylor algorithm, which are based on TDOA in UWB indoor positioning technology, were tested in Reference [35] on the basis of dynamic and static data in the case of an indoor line-of-sight (LOS) environment. The analysis results showed that decimeter-level positioning accuracy could be achieved using these tested localization methods. Moreover, the Taylor algorithm based on TDOA in UWB indoor positioning technology was able to achieve positioning accuracy of 1 decimeter. The work reported in Reference [36] presented a WLAN-based positioning system to locate the target devices through TDOA-based passive sniffers. The architecture, hardware, and algorithms for clock synchronization and system calibration were presented. The measurement results for the presented system provide a positioning error of 23 cm and 1.5 m for outdoor and indoor environments for WiFi with 80 MHz bandwidth.

Most of the previously reported TDOA-based localization systems provide the position of target node with certain level of accuracy. However, the capabilities of such systems are lacking when the same technology or system is also used for data collection (telemetry) along with the localization of the target node or sensor. The realization of such localization system with dual functionality could provide low-cost solutions in many applications. Moreover, such hybrid localization systems must be operated at low power so that they can be powered by batteries in order to obtain freedom of mobility for indoor measurements.

In this work, we used three ultra-low-power wireless systems (CC1101 radio transceivers configured in receive mode) from Texas Instruments as measuring receivers working at 434 MHz to locate the RF transmitter in 2D space. Instead of directly measuring the time differences for the localization of the RF transmitter, dc voltages are obtained by integrating the corresponding time difference pulses using an envelope detector, which effectively works as a pulse-width demodulator, and voltage level at fix time is measured from the resultant envelope detection signal. All three receivers are interfaced to the same computer using a CC debugger in order to achieve time synchronization. The same 434 MHz link can be used for telemetry in addition to localization capabilities by retrieving the telemetry data.

The contribution of the work presented here is three-fold. First, the implemented positioning system is based on very simple TDOA topology with reduced implementation complexity for effective localization of the target node with improved accuracy for both indoor and outdoor environments. Secondly, the presented localization system is low in cost, as it offers the additional capabilities of data collection from the target node along with its location information. The presented system has very low power requirements (less than 200 mW) due to the intrinsic low operating power characteristics of the three employed monitoring nodes. Finally, we improved the prediction by using machine learning techniques. We achieved the highest accuracy with Gaussian Process Regression (GPR) based on sample selection with differential entropy reduction criteria.

The rest of the paper is organized as follows: Section 2 describes the measurement principles for location sensing and the positioning algorithm based on TDOA technique that is used here for our proposed indoor positioning system. Section 3 explains the proposed TDOA-based indoor positioning system using ultra-low-power CC1101 radio transceivers from Texas Instruments. Section 3 provides the design, hardware implementation details and integration of different modules for the proposed TDOA-based localization system. In Section 4, the test setup and measurement results for the proposed system are presented for 2D localization, and the accuracy of system is determined by comparing the estimated location and the actual location of the wireless transmitter. The capability of the implemented system to receive telemetry data @ 250 Kbauds is also demonstrated in this section. Finally, we draw conclusions in Section 5.

## 2. Materials and Methods

For indoor localization, it is necessary to estimate the distance between the moving transmitter and the measuring receivers that are placed at known locations. This distance between the mobile transmitter and the measuring receivers is directly proportional to the propagation time. The basic idea of TDOA is to estimate the relative location of the moving transmitter by measuring the differences between the times at which the signal arrives at multiple receivers placed at known locations. TDOA-based localization techniques do not require time synchronization between the target transmitter and the measuring receivers; only between the time synchronization between the measuring receivers [27].

**Figure 1:**
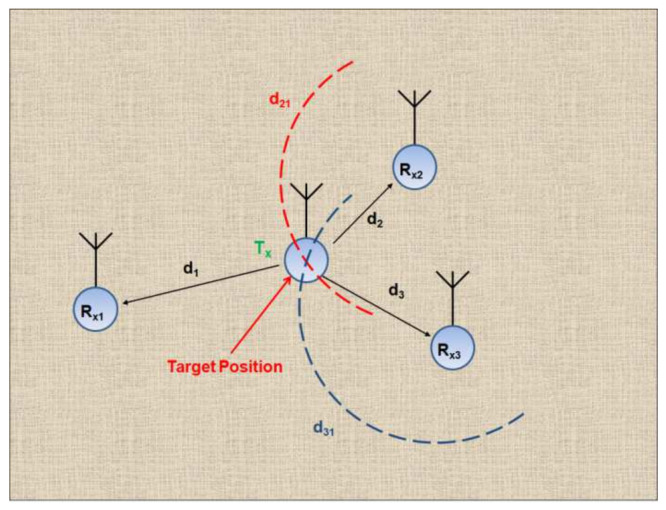
Illustration of the Time Difference of Arrival (TDOA)-based localization scheme.

Each TDOA measurement forms a hyperbolic curve in the localization space, which is used to estimate the position of the target transmitter. The intersection of multiple hyperbolic curves defines the possible position of the target. The position of the target transmitter in 2D can be estimated by deploying three measuring receivers [21], as shown in Figure 1. In the case of the 2D localization problem, assume the transmitter lies at (*x_o_, y_o_*) and the receivers are at (*x_i_, y_i_*) (*i* = 1, 2, 3) as shown in Figure 1. Then the distance differences can be given as [27]:

(1) $$d_{21}=d_{2}-d_{1}=\sqrt{{\left(x_{2}-x_{0}\right)}^{2}+{\left(y_{2}-y_{0}\right)}^{2}}-\sqrt{{\left(x_{1}-x_{0}\right)}^{2}+{\left(y_{1}-y_{0}\right)}^{2}}$$

(2) $$d_{31}=d_{3}-d_{1}=\sqrt{{\left(x_{3}-x_{0}\right)}^{2}+{\left(y_{3}-y_{0}\right)}^{2}}-\sqrt{{\left(x_{1}-x_{0}\right)}^{2}+{\left(y_{1}-y_{0}\right)}^{2}}$$

where *d_1_*, *d_2_* and *d_3_* represent the distance of the target transmitter from the respective receiver, with receiver 1 acting as the reference or common node for representation of distance differences. The two hyperbolic curves are formed from TDOA measurements related to three measuring receivers at known locations, and these curves are based on the above equations.

There should be some predefined conditions in order to estimate the correct location of the target transmitter using these equations, as two sets of solutions exist for these equations, corresponding to two foci. The accuracy of TDOA-based localization techniques is better than that of the Received Signal Strength Indication (RSSI)-based localization technique, as elaborated in [37], but they require time synchronization between the measuring nodes in order to perform position estimation [38]. We converted the TDOA to dc voltages as discussed in Section 3.1, and used machine learning techniques for location prediction. Initial predictions were obtained by the TLS algorithm and were further improved with GPR, Support Vector Machine (SVM), and Boosted and Bagged tree regression algorithms. All of the machine learning models were trained in MatLab2019b. The final highest accuracy results are based on the GPR technique. The GPR technique is a nonparametric method for the Bayesian approach, and instead of calculating a probability distribution over the parameters of a specific function, it utilizes the distribution of all admissible functions. A Gaussian Process (GP) represents a distribution over functions, where the distribution is defined by a mean *m*(*x*) and covariance *k(x, x′)*. Given a training set {(*x_i_,y_i_*); *I* = 1,2,…,*n*}, where *x*εR*^d^* represents an input vector and *y* ε R represents the response, a GPR model provides the response for the new input vector with the regression model of the form given in Equation (3):

(3) $$y=h{\left(x\right)}^{T}\beta +f\left(x\right)\;$$

where β is a constant, $h\left(x\right)$ represents explicit basis functions, *T* in superscript represents the transpose, and $f\left(x\right)$ is the function over the sampled data points *x*. The function *f*(*x*) is assumed to be distributed as a GP, and can be represented as shown in Equation (4):

(4) $$f\left(x\right)\sim GP\left(m\left(x\right),k\left(x,\;x^{\prime }\right)\right)$$

The mean of the GP,*m*(*x*), represents the expected value of the function, *m*(*x*) = *E*[*f*(*x*)] and covariance function *k*(*x*,*x′*) = *E*[(*f*(*x*) − *m*(*x*)) (*f*(*x′*) − *m*(*x′*))].

The response *y_i_* for the GPR model can be modeled using Equation (5), which represents the conditional probability distribution *p* for the response *y*:

(5) $$p(y|f\left(x\right),x)\sim N(y|h{\left(x\right)}^{T}\beta +f\left(x\right),\sigma^{2})$$

where N represents a normal distribution with variance $\sigma^{2}$. The covariance function *k*(*x*,*x′*) is also called the kernel function and is parameterized by hyper parameters. θ can also be written as *k*(*x*,*x′*| θ). Fitting the GPR model determines the parameters β, θ and variance $\sigma^{2}$. The GPR model is trained by an active selection of appropriate samples, providing the highest information gain to the model. The sample selection was based on the reduction in differential entropy. Given a random variable *Y* for response *y* and a random variable *X* for the input *x*, the differential entropy can be given for the response variable in Equation (6) as:

(6) $$H\left(Y\right)=-\int p\left(y\right)logp\left(y\right)dy$$

where *p*(*y*) represents the probability of the response y. The information gain for the selected input sample “*x*” can then be given by Equation (7), and input samples with higher information gain can be selected from the dataset to fit the GPR model.

(7) $$I\left(Y,X\right)=H\left(Y\right)-H\left(Y|X\right)=\int \left[-p\left(y\right)logp\left(y\right)dy+p\left(y,x\right)logp\left(y,x\right)\right]dy$$

The “fitrgp” Matlab 2019b function was used with sample selection criteria based on differential entropy GPR. The GPR model takes the voltage difference Vdc1, generated due to the time difference of arrival from receiver 1 and 2, and Vdc2, generated due to the time difference of arrival from receiver 1 and 3, to predict the location coordinates of the targets.

## 3. The Proposed Indoor Positioning System

### 3.1. Proposed System Architecture and Description

**Figure 2:**
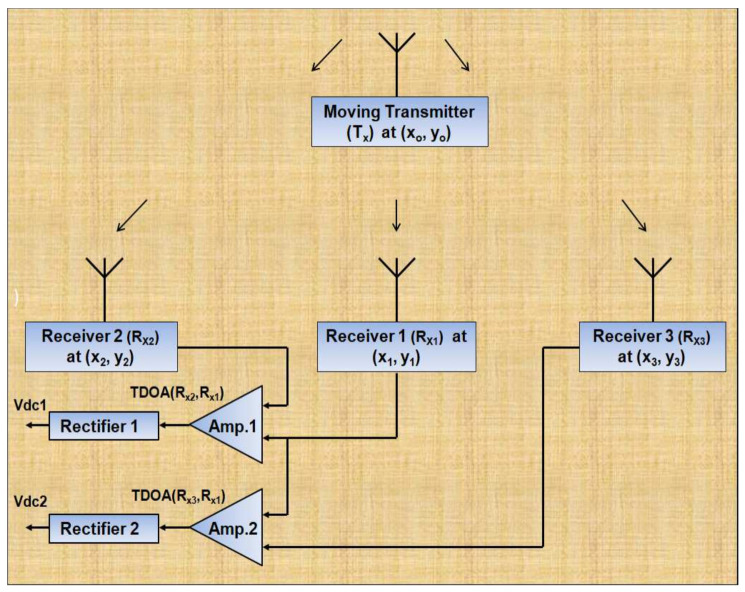
The architecture of the proposed TDOA-based positioning system using three receivers.

Our proposed TDOA-based indoor positioning system is based on three measuring receivers with one reference (common) receiver for TDOA measurements. The receivers were placed at (*x_1_*, *y_1_*), (*x_2_*, *y_2_*), (*x_3_*, *y_3_*), respectively, in order to be able to locate the moving target transmitter at (*x_0_*, *y_0_*) at any given instant, as shown in Figure 2. Three CC1101 ultra-low-power radio transceivers from Texas Instruments were used in receive mode as measuring units and one CC1101 device was used in transmit mode as the target transmitter for the testing and measurement setup. The CC1101 is a sub-1 GHz transceiver integrated with a configurable baseband modem to support various modulation formats (2-FSK, 4-FSK, GFSK, MSK, OOK, flexible ASK shaping), with a programmable data rate ranging from 0.6 to 600 Kbauds and a programmable output power of up to +12 dBm for all supported frequencies [39]. The CC1101 can be controlled via a serial peripheral interface (SPI). All three receivers were time synchronized by connecting them to the same computer. The transmitter was not required to be time synchronized with the measuring units, so it was interfaced with a separate computer system. Smart RF Studio was used to interface theCC110L with the computer through the CC debugger in order to perform its configuration. CC debuggers are primarily used for debugging and programming the System-on-Chip devices (SoC) from Texas Instruments; when connected to the debugger, SoC devices can be controlled directly from SmartRF™ Studio. SmartRF™ Studio communicates with the CC110L over the USB interface, and the debugger is powered by plugging the USB cable into the computer after connecting the debugger to the CC1101 transceiver [40]. The hardware and software requirements for the personal computer required to configure CC1101 devices are defined by SmartRF™ Studio.

As shown in Figure 2, the received data pulses (digital data output from respective receivers) from the three CC1101 measuring receivers are fed to a high-speed differential circuit to extract the time differences required for the TDOA mechanism. Two high-speed AD8132 (3 dB bandwidth of 350 MHz) and low-power (10.7 mA @ 5 V) differential amplifiers were used to measure Δt1 (time difference between the signals received by R_x2_ and R_x1_) and Δt_2_ (time difference between the signals received by R_x3_ and R_x1_). The time difference signals from the differential circuits are demodulated (rectified) using the envelope detector and sampled to extract the dc voltages corresponding to Δt_1_ and Δt_2_, which can be mapped to distance differences to locate the moving target transmitter, as shown in Figure 2 for the proposed system.

### 3.2. Proposed System Design, Implementation and Integration

**Figure 3:**
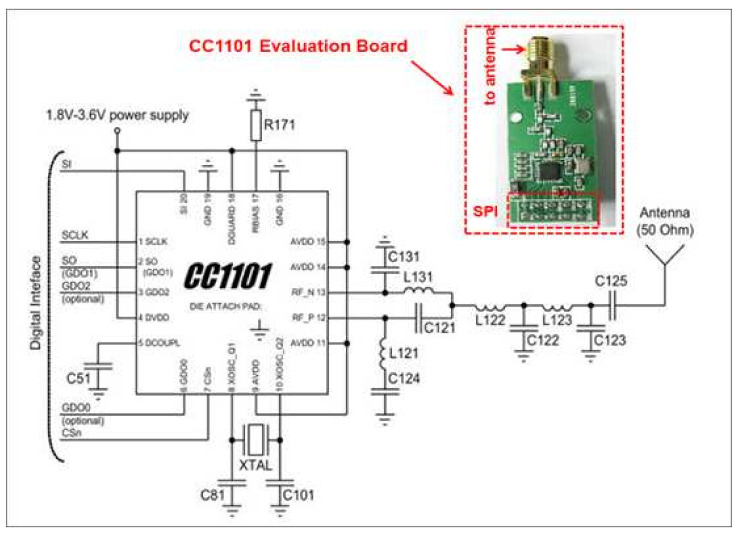
The CC1101 circuit schematic for 434 MHz operation and its evaluation board [30].

The proposed localization system was implemented using three CC1101 devices (configured in receive mode) as measuring units and one CC1101 device as the target transmitter (configured in transmit mode). Four evaluation boards from Texas Instruments were used for this purpose. The circuit schematic of CC1101 for 434 MHz and its evaluation board are shown in Figure 3. The CC1101 evaluation board was configured (T_x_ or R_x_ mode, modulation type, data rate, operating frequency, transmit power for T_x_ mode, etc.) using SmartRF™ Studio, which communicates with CC1101 through SPI via a CC debugger, as discussed earlier.

**Figure 4:**
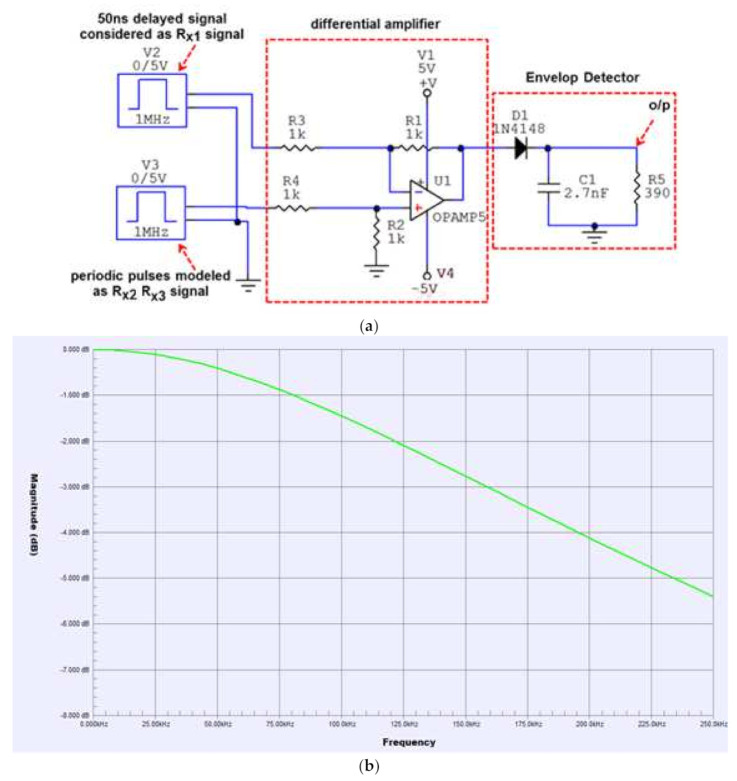
(**a**) Circuit Maker schematic for the differential amplifier and the envelop detector. (**b**) The frequency response of the circuit presented in (**a**).

**Figure 5:**
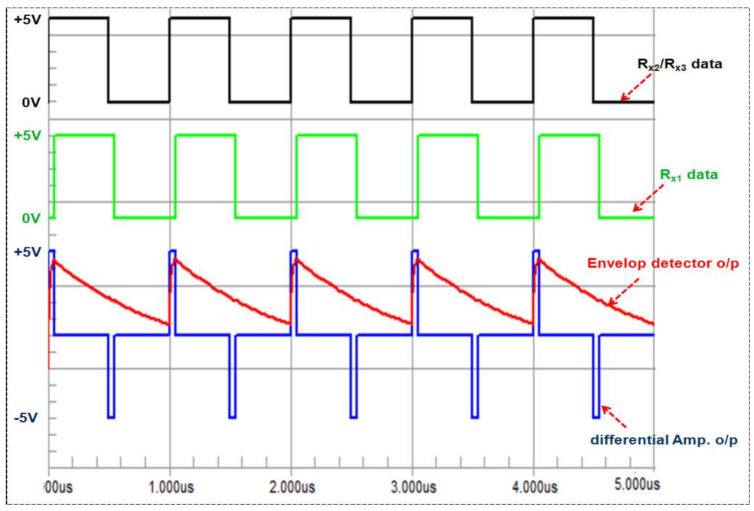
The Circuit Maker simulation results for the differential amplifier and envelop detector.

The differential amplifier circuits are fed by the received digital data pulses from the respective receivers in order to generate the corresponding time difference pulses, which act as the input to the envelope detector for pulse width demodulation (rectification), as shown in the schematic (Figure 4a). These periodic pulses are from the result of time delays between the two signals received by the respective receivers. One time pulse corresponds to a time difference at the rising edges and other one with opposite polarity from the falling edges of the two received signals, as can be clearly seen in the Circuit Maker simulation results shown in Figure 5. The envelope detector is also shown in Figure 4a at the output of the differential amplifier circuit. It consists of a diode and an RC circuit, and discards the negative valued time difference pulses due to the diode in the patch, as shown in Figure 5 on the basis of simulation results for the differential amplifier and the envelope detector circuit.

Although all three receivers were connected to same laptop/computer, they were operated by a 26 MHz internal clock provided by the crystal oscillator of the CC1101 transceiver, so there will be clock jitter for each time difference pulse. The envelope detector effectively works as a pulse-width demodulator, and is insensitive to clock jitter due to its low bandwidth. In order to configure the CC1101 in receive mode, it has a programmable channel-filter bandwidth ranging from 58 KHz to 812 KHz.

The bandwidth of the envelope detector was calculated using Equation (8), given below:

(8) $$f_{3dB}=\frac{1}{2*\pi *Timeconstant\left(RC\right)}=\frac{1}{2*\pi *2.7nF*390\;Ω}\;f_{3dB}=0.15\;\mathrm{MHz}\;$$

**Figure 6:**
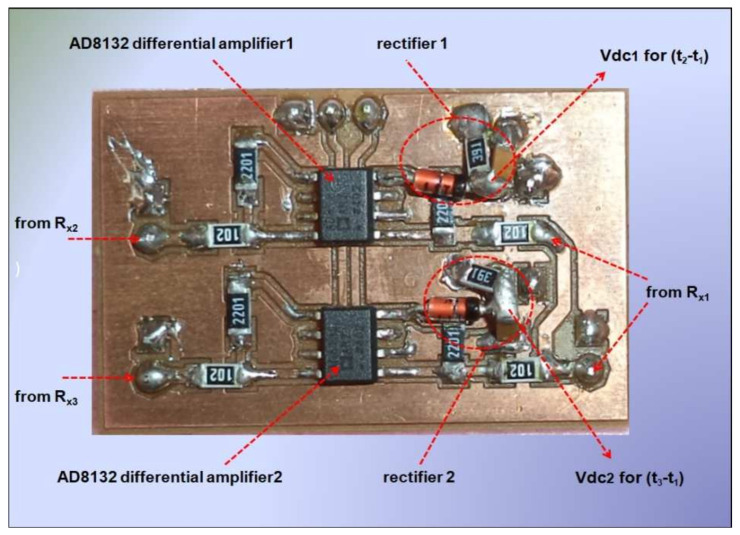
The implemented differential amplifiers with the envelope detector.

As the bandwidth of envelope detector (15 MHz) is low compared to the bandwidth of the differential amplifier, the effect of jitter is minimized, and the envelope detector can effectively provide stable voltage levels corresponding to the time difference of arrival, which is used for the localization of the target transmitter. This fact is also confirmed through the simulated frequency response in Figure 4b for the complete circuit (differential amplifier + envelop detector). As can be seen from Figure 4b, the 3 dB bandwidth of the complete circuit is restricted to 0.160 MHz or 160 KHz due to the envelope detector. The two high-speed AD8132 (350 MHz) and low-power differential amplifier circuits with envelop detectors were implemented on a single FR-4 board to measure Vdc1 and Vdc2, as shown in Figure 6.

**Figure 7:**
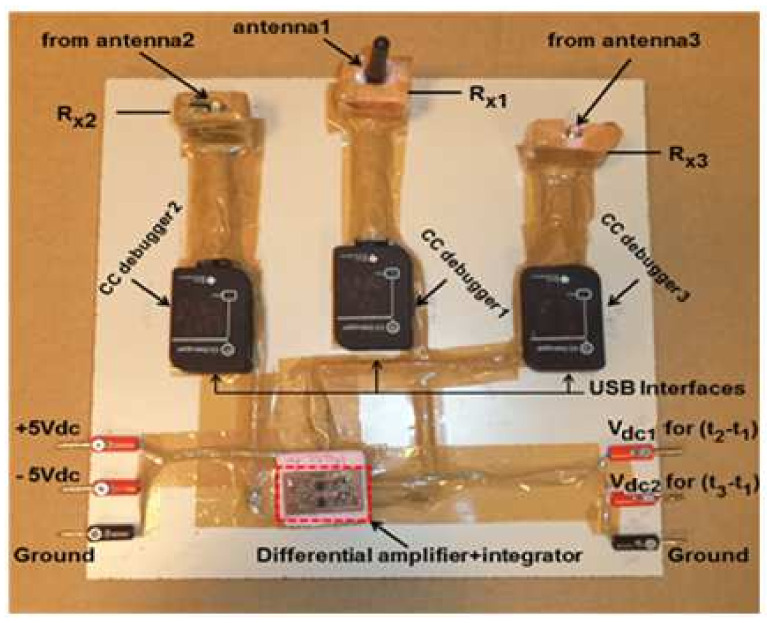
The implemented TDOA-based IPS system using three TI CC1101 radio transceivers.

The compact implemented indoor positioning system (IPS) is shown in Figure 7, where all the components and modules were integrated and fixed on a single wooden board containing dc power supply connection, output interface, USB interfaces, etc.

## 4. Results and Discussion

**Figure 8:**
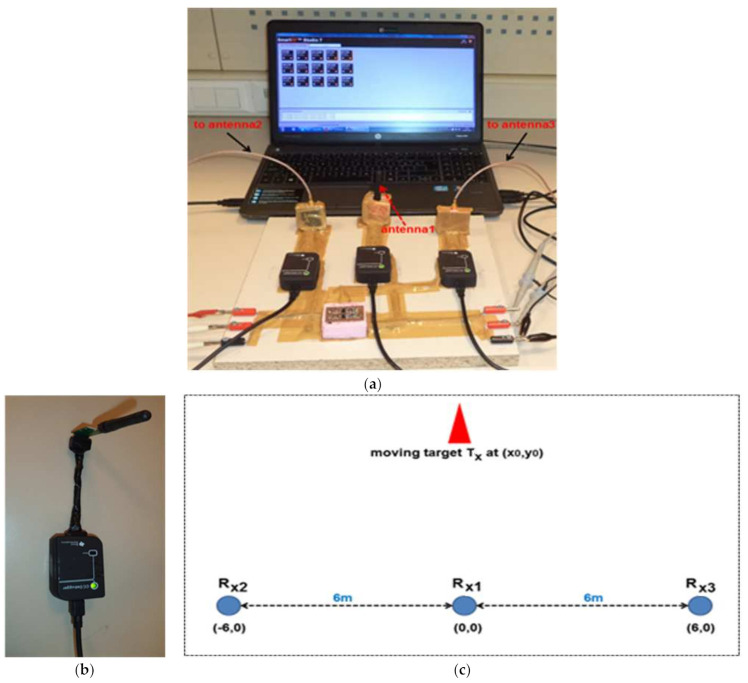
Test and measurement setup: (**a**) three CC1101 as receivers; (**b**) one CC1101 as transmitter; (**c**) placement of the three measuring receivers in 2D space to locate the target T_x_.

The implemented IPS was tested both for indoor and outdoor wireless environments. To perform the measurements, the IPS was connected to the same laptop via USB interfaces for the configuration and time-synchronized operation of the three measuring receivers, as shown in Figure 8a. The CC1101, configured as the target transmitter, is shown in Figure 8b, and was connected to a second laptop placed on a moveable test and measurement bench in order to be able to change the location or position of the transmitter for testing purposes. The transmission power of the target transmitter was appropriately configured in order to establish a reliable wireless communication link between the transmitter and receiver nodes.

Although three receivers were fixed on same board, the antennas of receiver 2 and receiver 3 were spatially displaced with respect to receiver 1, which was used as the common node for measuring (d_2_-d_1_) and (d_3_-d_1_). The antennas for both R_x2_ and R_x3_ were placed 6 m away from R_x1_ along the horizontal axis and connected to respective receivers through cables of equal length. If the location of R_x1_ in 2D space is assumed to be (0, 0), then R_x2_ was located at (−6, 0) and Rx3 was positioned at (6, 0), as depicted in Figure 8c Thus, with reference to Figure 2 and Figure 8c, the co-ordinates of the measuring receivers were:

(9) $$(x_{1}\;,y_{1})=\left(0\;,\;0\right),(x_{2\;},y_{2})=\left(-6\;,\;0\right),(x_{3}\;,y_{3})=\left(6\;,\;0\right)$$

**Figure 9:**
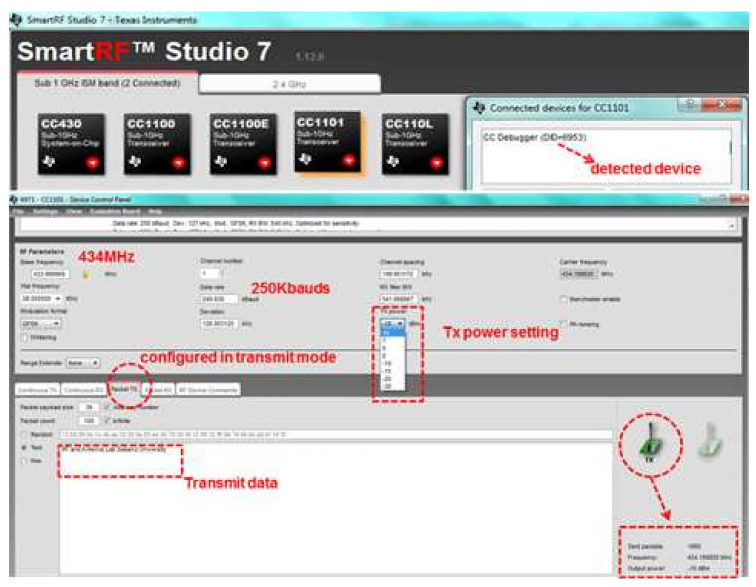
The CC1101 configured as a 434 MHz transmitter (−10 dBm, 250 Kbaud data rate).

The target transmitter was configured using SmartRF™ Studio to perform continuous packet transmission at a carrier frequency of434 MHz with −10 dBm transmission power. The data rate was set to 250 Kbauds for the continuous transmission of a text message using GFSK modulation. The configuration of the target transmitter’s parameters using the SmartRF™ Studio interface is presented in Figure 9.

**Figure 10:**
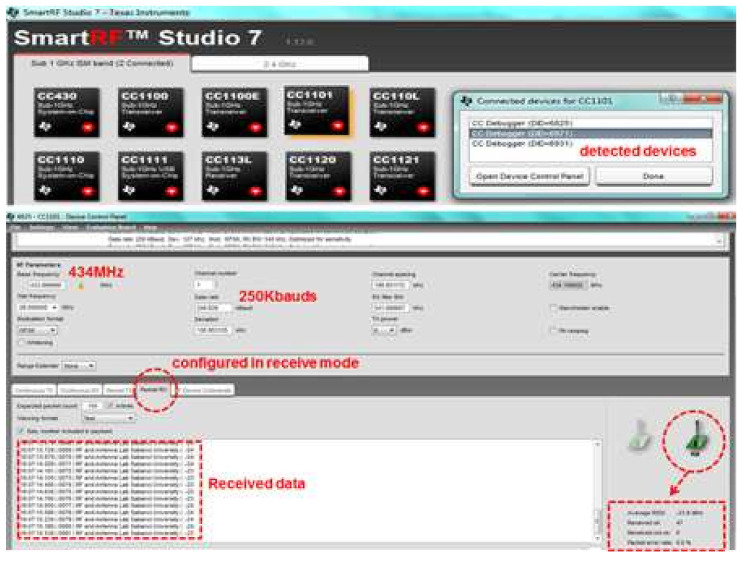
Three CC1101devices configured as 434 MHz measuring receivers.

Similarly, three measuring receivers of the implemented localization system were also configured using SmartRF™ Studio to receive continuous packets at a carrier frequency of 434 MHz and a data rate of 250 Kbauds using the GFSK demodulation format (used as one option for optimized Rx sensitivity). The configuration of parameters using SmartRF™ Studio for the measuring receiver interface is illustrated in Figure 10.

To analyze the performance of the wireless telemetry link, the transmitter was triggered to continuously transmit packets for the text message while three measuring receivers were started in order to receive the telemetry data, as shown in Figure 9 and Figure 10, respectively. The SmartRF™ Studio interface for receiving operations provided the telemetry link performance parameters on the basis of the Received Signal Strength Indication (RSSI), and number of error-free and number of corrupted packets received, as shown in Figure 10. As can be seen in Figure 10, the 250 Kbaud telemetry data link working at 434 MHz exhibited satisfactory performance, as it continuously received text data with error-free packets with good RSSI. According to the requirements of the remote telemetry transmitting node, the telemetry receiver’s data rate can be configured to between 0.6 to 600 Kbauds by using different demodulation formats (2-FSK, 4-FSK, GFSK, MSK, OOK, etc.).

To address the localization problem, the system was tested both in outdoor and indoor environments in order to analyze its performance for LOS and NLOS scenarios, respectively. The transmitter was initially placed at (*x_0_*, *y_0_*) = (0, 15) with reference to Figure 8c in order to calibrate the system. In this case, T_x_ was located at equal distance from both R_x2_ and R_x3_, which should provide almost the same amount of measured dc voltage.

**Figure 11:**
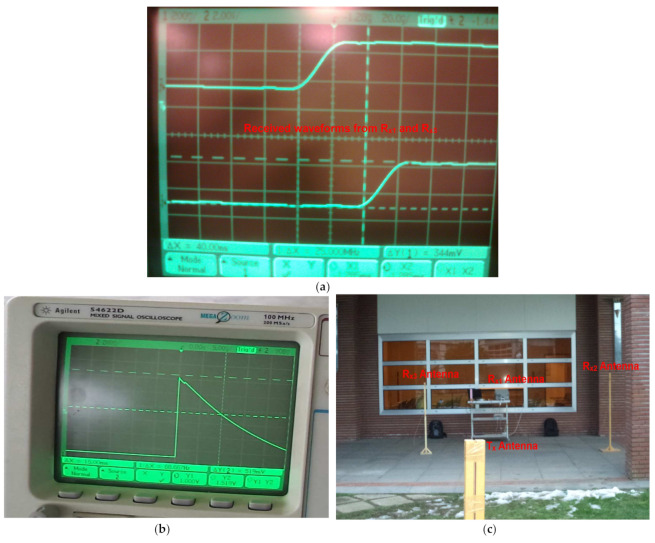
The oscilloscope screenshots of received waveforms from two receivers and corresponding output of envelope detector along with measurement setup.(**a**) received waveforms from R_x1_ and R_x3_; (**b**) output of the envelope detector; (**c**) test and measurement setup.

**Figure 12:**
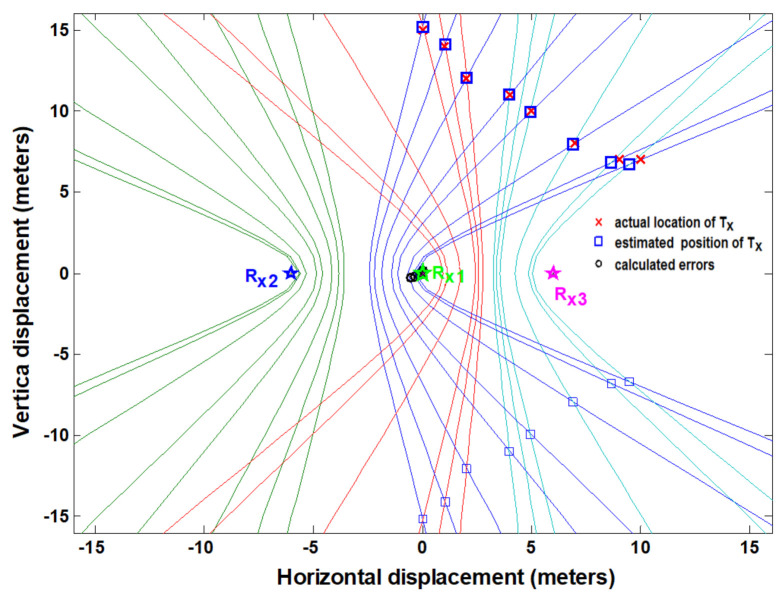
The estimated (measured) location vs. the actual location of the target transmitter in an LOS environment (outdoor scenario).

For the outdoor localization case (LOS scenario), the measured dc values, i.e., Vdc1 and Vdc2, are shown in Table 1. These measurements were performed in an open environment with no obstacles present between the target node and the monitoring units (three receivers), as can be clearly seen in Figure 11. The measured dc values correspond to time differences (t_2_-t_1_) and (t_3_-t_1_), respectively, where t_1_, t_2_ and t_3_ are the required propagation times for the transmitted signal to reach their respective receivers. These time differences are ultimately related to distance differences (d_21_ and d_31_). Although the implemented system provides the absolute dc values for corresponding displacements, the polarity of the measured dc values can be predicted by tracking the previous dc values. For example, when the target transmitter is moved from (0, 15) to (2, 12), the measured V_dc1_ increases, but the V_dc2_ decreases, as the transmitter is moving away from R_x2_ and approaching R_x3_. On the other hand, when Tx is positioned at (4, 11),absolute V_dc1_ increases, while absolute V_dc2_ also increases, rather than decreasing, so the polarity of V_dc2_ is inverted, as highlighted in Table 1. The position of the target transmitter is estimated by plotting the time difference hyperbolic curves using the measured dc values. The results for computed location versus the actual location of target transmitters placed at different positions in the LOS environment are shown in Figure 12. The localization error distance is also computed by taking the difference between the measured and actual positions of T_x_ in both dimensions. The maximum computed error was 0.8 m in both dimensions (*X* and *Y*). The oscilloscope screenshots of the received waveforms from the two receivers and the corresponding output of the envelope detector are also shown in Figure 11. The maximum localization size is limited by the transmit power of the target transmitter and the sensitivity of the employed receivers. Meanwhile, the minimum localization size and resolution of the localization are defined by the bandwidths of the differential amplifier and the envelope detector.

**Figure 13:**
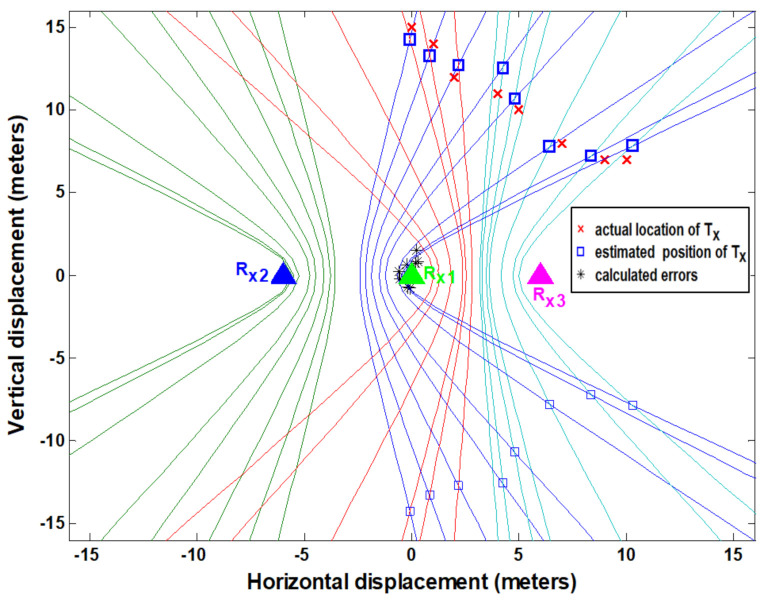
The estimated (measured) location vs. actual location of target transmitter in NLOS environment (indoor scenario).

In the case of indoor measurements (NLOS case), the measured Vdc1 and Vdc2 values are shown in Table 2. Again, the polarity of the measured value is determined as discussed for the LOS measurement case. The time difference contours are plotted using the measured dc values for (t_2_-t_1_) and (t_3_-t_1_). The position of the target transmitter is extracted on the basis of the intersection points of two hyperbolic curves, as shown in Figure 13. The localization error distances are again computed by taking the difference between the extracted and actual positions of T_x_ in both dimensions (*X* and *Y*). The maximum computed errors were 0.8 m and 1.5 m in the *X* and *Y* dimensions, respectively. The positioning accuracy decreases for indoor measurements due to multipath effects. These indoor localization measurements were performed inside the building, with the target node (target transmitter) placed outside the lab and monitoring units arranged inside the lab. In this case, the NLOS scenario was established by the presence of concrete walls between them. This also validates the through-wall performance of the system.

The prediction is further improved by using machine learning techniques in MatLab for the prediction of coordinates on the basis of the voltage values vdc1 and vdc2. This technique uses SVM, GPR, Boosted tree and Bagged tree ensemble for the prediction of coordinates, as illustrated in Table 3.

The lowest error rates of 0.68 m and 1.08 m for LOS and NLOS cases were given by the GPR, followed closely by the linear regression method we previously used in our analysis. SVM gave a slightly higher error than linear regression. However, both the tree ensemble algorithms including boosted and bagging trees had much higher error, as illustrated in Table 3.

A comparison of the performance achieved by the proposed IPS with that of previously proposed systems is given in Table 4. The proposed design has the highest accuracy of 0.8 m for LOS and 1.5 m for NLOS with linear regression, and 0.68 m and 1.08 m for LOS and NLOS are achieved with GPR. It also provides low-cost, simple design and low dc power consumption. Furthermore, the proposed system provides dual functionality of both localization and configurable data telemetry over the same link.

## 5. Conclusions

In this work, a 434 MHz TDOA-based indoor positioning system for smart homes was introduced. The positioning system uses machine learning techniques to achieve the highest accuracy for existing systems. The highest accuracy was achieved with GPR based on sample selection with a reduction in differential entropy. The proposed system uses ultra-low-power (less than 200 mW) wireless systems from Texas Instruments with three measuring receivers, which were deployed and tested for 2D applications. The inference process is based on a limited set of eight samples covering the position coordinates in the room, and is not computationally intensive. For new voltage values, there are eight subtractions and seven sums of products. The operations are low in cost and consume very low power in most modern energy-efficient microcontrollers, e.g., the modern ARM M0 cores consumes a single cycle for MUL operation and consume a pJ of power per cycle [46]. The power consumption of the computations involved in the inference is much lower than the 200 mW consumed by the system. The time difference pulses were integrated or demodulated using the envelope detector to minimize the error caused by clock jitter. The implemented system achieved a positioning accuracy of0.68 m and 1.08 m for outdoor and indoor localization scenarios, respectively. The implemented system achieved improved positioning accuracy when compared to previously reported positioning systems [13,32,33,34]. The system additionally has the ability to receive telemetry data, and was tested with250Kbauds as the data rate, but the data rate can be configured to anywhere between 0.6 and600 Kbauds with different demodulation formats. The performance of the presented localization system can be further improved by deploying envelope detection circuitry with ability to measure negative valued pulses in order to automatically determine the polarity of the measured dc values, thus achieving better localization accuracy. Future work could be performed in order to improve the localization accuracy of the presented system for NLOS environments. Moreover, the differential amplifier can be replaced with an instrumentation amplifier in order to reduce system noise and make it less sensitive to the temperature and tolerances of the passive components used in differential circuitry.

Our future work consists of further performing a deeper mathematical analysis of hybrid methods and corresponding CLRB performance. In future work, the CRLB will be adapted to our model and models of any dependent parameter variances will need to be included in the computation of the inverse of the Fisher Information Matrix. We further plan to add a hybrid technique and evaluate a hybrid CLRB.

## Figures and Tables

**Table 1:** Measured dc values with target positioned in Line of Sight (LOS) at different locations.

T_x_ Position (*x*,*y*)	Vdc1 (mV) (d_2_-d_1_)	Vdc2 (mV) (d_3_-d_1_)
(0,15)	115	116
(1,14)	162	83
(2,12)	226	48
(4,11)	316	−52.5
(5,10)	369	−113
(7,8)	463	−257
(9,7)	515	−378
(10,7)	526	−414

**Table 2:** Measured dc values with target positioned in Non-Line of Sight (LOS) at different locations.

T_x_ Position (*x*,*y*)	Vdc1 (mV) (d_2_-d_1_)	Vdc2 (mV) (d_3_-d_1_)
(0,15)	123.5	131
(1,14)	170	100
(2,12)	233	40
(4,11)	310	−60.5
(5,10)	365	−100
(7,8)	480	−242
(9,7)	528	−363
(10,7)	540	−420

**Table 3:** Machine learning prediction for target coordinates.

Machine Learning Algorithm	LOS Error (m)	NLOS Error (m)
Total Linear Regression	0.80	1.50
Gaussian Process Regression	0.68	1.08
Support Vector Machine (SVM)	0.89	1.63
Boosted Trees Ensemble	6.93	7.31
Bagged Trees Ensemble	6.95	7.20

**Table 4:** The performance comparison of the presented IPS with previously reported systems.

IPS Ref.	Tech.	Accuracy	Advantage	Disadvantage
[13]	Angle of Arrival (AOA)	2.5 m	Employs two access-points for positioning	Infrastructure cost would be much higher. Mainly for the line of sight (LOS)-dominating environments.
[41]	Time Difference of Arrival (TDOA)	≤2 m	Improvd accuracy of sub-meter level	Two-step algorithm which require additional computing
[42]	Received Signal Strength Indication (RSSI)	2 m	Automatic generation and calibration of the fingerprinting database	Accuracy dependent on path loss parameters
[43]	Plane model position	3 m	Reduced computing complexity	The variations in signal strength due to obstacles were not considered.
[44]	RSSI	2 m	Simple algorithm	Low localization range
[45]	RSSI	1.18	Improved positioning accuracy	The accuracy is affected by received signal variations
Proposed Indoor Positioning System (IPS)	TDOA	0.68 m for LOS 1.08 m for non-line of sight (NLOS)	Simple design with good accuracy and low DC power. Dual functionality of localization and telemetry.	Lower accuracy for NLOS environment

## Data Availability

Data is contained within the article.
